# Reconstruction of damaged cornea by autologous transplantation of epidermal adult stem cells

**Published:** 2008-06-05

**Authors:** Xueyi Yang, Nicanor I. Moldovan, Qingmei Zhao, Shengli Mi, Zhenhui Zhou, Dan Chen, Zhimin Gao, Dewen Tong, Zhongying Dou

**Affiliations:** 1 Department of Life Sciences, Luoyang Normal University, Luoyang, China; 2 Shaanxi Branch of National Stem Cell Engineering Center, Northwest A & F University, Yangling, China; 3 Davis Heart and Lung Research Institute, Ohio State University, Columbus, OH,; 4 Department of Animal Science and Veterinary, Beijing Agricultural Vocation College, Beijing, China; 5 Electron Microscopic Center, School of Basic Medicine, Fourth Military Medical University, Xi’an, China

## Abstract

**Purpose:**

It is crucial for the treatment of severe ocular surface diseases such as Stevens-Johnson syndrome (SJS) and ocular cicatricial pemphigoid (OCP) to find strategies that avoid the risks of allograft rejection and immunosuppression. Here, we report a new strategy for reconstructing the damaged corneal surface in a goat model of total limbal stem cell deficiency (LSCD) by autologous transplantation of epidermal adult stem cells (EpiASC).

**Methods:**

EpiASC derived from adult goat ear skin by explant culture were purified by selecting single cell-derived clones. These EpiASC were cultivated on denuded human amniotic membrane (HAM) and transplanted onto goat eyes with total LSCD. The characteristics of both EpiASC and reconstructed corneal epithelium were identified by histology and immunohistochemistry. The clinical characteristic of reconstructed corneal surface was observed by digital camera.

**Results:**

Ten LSCD goats (10 eyes) were treated with EpiASC transplantation, leading to the restoration of corneal transparency and improvement of postoperative visual acuity to varying degrees in 80.00% (8/10) of the experimental eyes. The corneal epithelium of control groups either with HAM transplantation only or without any transplantation showed irregular surfaces, diffuse vascularization, and pannus on the entire cornea. The reconstructed corneal epithelium (RCE) expressed CK3, CK12, and PAX-6 and had the function of secreting glycocalyx-like material (AB-PAS positive). During the follow-up period, all corneal surfaces remained transparent and there were no serious complications. We also observed that the REC expressed CK1/10 weakly at six months after operation but not at 12 months after operation, suggesting that the REC was derived from grafted EpiASC.

**Conclusions:**

Our results showed that EpiASC repaired the damaged cornea of goats with total LSCD and demonstrated that EpiASC can be induced to differentiate into corneal epithelial cell types in vivo, which at least in part correlated with down-regulation of CK1/10 and upregulation of PAX-6.

## Introduction

The integrity of the normal ocular surface depends on its epithelial stem cells located at the limbus, termed limbal stem cells (LSCs). LSCs serve as the ultimate source for maintenance and regeneration of the corneal epithelium [1-3]. Damage to the limbus can result in limbal stem cell (LSC) deficiency, characterized by conjunctivalization, vascularization, and chronic inflammation (reviewed in Espana et al.) [4]. Transplantation of cultivated LSCs for reconstructing the ocular surface of total LSC deficiency is one recently developed treatment [5-8]. Damaged corneas have been successfully reconstructed by transplanting autologous limbal epithelial cells [7,8] but not in severe ocular surface diseases such as Stevens-Johnson syndrome (SJS) and ocular cicatricial pemphigoid (OCP). In the case of severe ocular diseases, the bilateral damage to LSCs and the immune response to heterologous corneal epithelial transplantation are the most challenging clinical entities. Therefore, our goal was to examine whether epidermal stem cells possess the capacity to activate corneal genetic programs in response to corneal stromal stimuli.

It is widely accepted that epidermis and corneal epithelium are derived from ectoderm during embryogenesis. A comparison of the corneal epithelium and follicular epidermis illustrates that keratinocyte stem cells of these two systems share several important attributes (reviewed in Taylor et al.) [9]. These two cell types express the same markers such as protein 63 (P63), β1-integrin, and cytokeratin 19 (CK19) [10-12]. Ferraris et al. and Pearton et al. [13,14] have shown that the transient amplifying (TA) cells of the central corneal epithelium can be converted into the epidermis and its appendages. This reprogramming occurs by means of a multi-step process that involves the de-differentiation of TA cells into primitive stem cells followed by re-differentiation into hair follicles and associated multipotent stem cells [14]. Liang and Bickenbach [15,16] have demonstrated that somatic epidermal adult stem cells (EpiASC) have the ability to produce cells of different lineages during development and suggested that EpiASC may have pluripotentiality similar to embryonic stem cells (ESC). These authors co-cultured EpiASC with embryonic stem cells or transfected keratinocytes with Octamer-4 (Oct4) and found that EpiASC could alter their cell lineage protein expression to that of a more primitive cell type [17,18]. Thus, the microenvironment of the organ’s structure induces cells of various origins and degrees of stemness to undergo a 'functional adaptation' to fit into the overall role of the host organ [19]. This implies that EpiASC might convert into corneal cell types when recombined by the corneal stroma in vivo and also suggests that transplantation of EpiASC may be a good way of solving the clinical problems encountered in severe ocular diseases.

It is well known that EpiASC in vivo are harbored in niches, which keep them in a state of quiescence and stemness [20-22]. EpiASC are surrounded by a group of TA cells, which become active and proliferate in culture [23,24]. TA cells may play an important role in keeping EpiASC quiescent and in protecting them from damage [9,25,26]. However, purification of EpiASC is still problematic because of the absence of a reliable, specific marker. Many researchers have reported the isolation of EpiASC from the skin by digesting the explants with enzymes such as trypsin and dispase (reviewed in Kaur et al.) [27]. To address this purification problem, we used a recently developed strategy to isolate and purify EpiASC [28]. To identify whether EpiASC can convert into corneal cell types when recombined by the corneal stroma in vivo, we developed a method of culturing EpiASC on denuded human amniotic membrane (HAM) and then transplanted the resulting autologous EpiASC-HAM sheet (EHS) onto the ocular surface of a goat model of limbal stem cell deficiency (LSCD). The clinical outcome was assessed after 30 months of follow up [29].

## Methods

### Isolation and identification of epidermal stem cells from the goat ear skin

The isolation and identification of epidermal stem cells were performed as previously described [28]. Briefly, pieces of skin approximately 0.5 cm × 0.6 cm were cut from the ear of Guanzhong dairy goats (three to four years old). The tissues were rinsed three times with Medium 199 (Invitrogen-Gibco BRL, Grand Island, NY), which contained 50 μg/ml gentamicin and 1.25 μg/ml amphotericin B. After the careful removal of cartilage and conjunctive tissue, the remaining tissue was moved to a culture dish and cut into pieces approximately 1 mm × 2 mm × 3 mm using a scalpel. We then placed three to four pieces of the explant into each well of a six-well TC-Plate (Hyclone, Logan, UT) and added Medium 199 (Invitrogen-Gibco BRL, Grand Island, NY) supplemented with 15% neonatal bovine serum (Baian Biotechnology Corporation, Zhengzhou City, China), 5 μg/ml insulin (Sigma-Aldrich Corporation, St. Louis, MO), 0.5 μg/ml hydrocortisone, 40 μg/ml gentamicin, and 0.25 μg/ml amphotericin B. The explants were cultured at 37 °C in a 5% CO_2_ /95% air incubator. The medium was changed every two to three days while the extent of each outgrowth was monitored with a phase contrast microscope. We selected the holoclones under dissecting microscope and treated them with 0.2% trypsin and 0.02% EDTA for approximately 10 min to disperse each clone to single cells. The trypsin was then inactivated by adding a medium containing 20% serum, and cells were gently centrifuged and resuspended in a serum free medium. The selected cells were seeded into culture dishes coated with gelatin type B (Sigma) in a medium containing 80% Ham F12 (Invitrogen-Gibco BRL, Grand Island, NY), 20% GSFCM (goat skin fibroblast-conditioned medium), 1 - 2% BSA (Sigma-Aldrich, St. Louis, MO), 20 ng/ml EGF (Sigma), 20 ng/ml IGF-1 (Sigma), 5 μg/ml insulin, 0.5 μg/ml hydrocortisone, and 50 μg/ml penicillin and streptomycin.

Primary putative epidermal stem cells were left to grow until they reached about 70% confluence in the culture plate. Adherent cells were then harvested with 0.2% trypsin and 0.02% EDTA to dislodge the cells followed by rapid trypsin neutralization to prevent cell damage. Isolated single cell suspensions were subcultured on culture dishes coated with gelatin at final plating density of 0.5–2 × 10^5^ viable cells per well. At each passage, cells from one well out of a set of triplicate cultures were detached, pooled, centrifuged, resuspended in fresh growth medium, seeded into three new wells, and expanded until they reached the same level of confluence. The growth medium was changed completely every other day. The cultures were monitored under a Leica Microsystems Wetzier microscope, LEICA MPS 60 (Leitz, Wetzlar, Germany).

To confirm the EpiASC derived from interfollicular epidermis, we dissected epidermis from dermis as previously described [30] with the following modifications. Skin samples were treated with 0.5% dispase II (Boehringer, Ingelheim, Germany) for 14–16 h at 4 °C to separate the interfollicular epidermis as a sheet from the underlying dermal tissue, leaving the hair follicular from the infundibulum to the dermal papillae within the dermis. The dermal surface was brushed gently with curved forceps to release any loosely attached basal keratinocytes, and then both interfollicular epidermis and dermis were cultured in the same medium. The cultures were monitored under a Leica Microsystems Wetzier microscope LEICA MPS 60 (Leitz Wetzlar).

### Preparation of human amniotic membrane and cultivation of autologous epidermal stem cells on human amniotic membrane

Human amniotic membranes (HAMs) were obtained at the time of Cesarean section with proper informed consent in accordance with the tenets of the Declaration of Helsinki for research involving human subjects and upon approval by the Institutional Review Board of the Northwest A&F University (Shaanxi, Yangling, China). Under sterile conditions, the membranes were washed with sterile phosphate-buffered saline (PBS) containing antibiotics (200 IU/ml penicillin and 200 IU/ml streptomycin) then were incubated with 2.5% dispase at 4 °C for 12–20 h to loosen cellular adhesion followed by gentle scraping with a cell scraper and washing three times with PBS. The denuded HAM was frozen and stored at −80 °C in pure glycerol. Immediately before use, the HAM was thawed, washed three times with PBS containing antibiotics, cut into approximately 4 cm x 4 cm pieces, and flattened with the stromal side on the surface of a nitrocellulose paper (NCP) framework.

We used passage three to four putative EpiASC as seed cells. The EpiASC (1 × 10^5^ cells/ml) were seeded on the denuded HAM spread upon the NCP frame in a culture dish. The culture was submerged in a serum-free medium as described above for one week and then exposed to air by lowering the medium level over a two week period. The medium contained 80% F12, 20% GSFCM, 1%–2% BSA, 20 ng/ml EGF, 20 ng/ml IGF-1, 5 μg/ml insulin, 0.5 μg/ml hydrocortisone, 50 μg/ml Vitamin C (Sigma), and penicillin and streptomycin (50 μg/ml each). Cultures were incubated at 37 °C in a 5% CO_2_/95% air incubator for up to 21 days, and the medium was changed every day.

### Creation of total limbal stem cell deficiency model

The model of total LSCD was created in the left eye of 30 goats using a method previously described [28] with slight modifications. Briefly, after anesthesia, an ocular surface injury was created in one eye of each of the 26 healthy Guanzhong dairy goats by excising all the conjunctival tissue with 5–7 mm of the limbus and performing a superficial keratectomy of the central corneal surface including limbal epithelial cells, and then the limbus was burned by mechanical erasure with 1 N NaOH. The goats received chloramphenicol and dexamethasone eye drops twice a day during the first week and the necessary care for 28 days after surgery. LSCD was verified using clinical signs such as corneal haze, vascularization, epithelial irregularity, and the presence of conjunctival goblet cells on the corneal surface.

### Transplantation of autologous cultivated epidermal stem cells on human amniotic membrane sheets

Four weeks after the ocular surface injury, the conjunctivalized ocular surfaces of 10 goats were surgically reconstructed by receiving transplants of EpiASC-HAM sheets (EHS), eight animals received HAM only transplantation (first control group), and the remaining eight underwent removal of the fibrovascular pannus without any transplantation (second control group) [28]. After surgery, the eye was covered with sterile gauze. From the first postoperative day, the goats were treated with topical preservative-free 1% methyl prednisolone acetate and 0.3% ofloxacin four times a day and with intramuscular penicillin sulfate and streptomycin sulfate every day for a week.

**Table 1:** Primary antibodies and sources

**Antibodies**	**Category**	**Dilution**	**Source**
CK1/10	Mouse monoclonal	50X	Abcam®.
PAX-6	Mouse monoclonal	1000X	Chemicon®
CK3	Mouse monoclonal	50X	USBiological
CK12	Mouse monoclonal	100X	Santa Cruz Biotechnology, Inc.
P63	Mouse monoclonal	200X	Santa Cruz Biotechnology, Inc.

The primary antibodies used in this study and their sources.

### Histology and immunohistochemistry

To identify whether epidermal stem cells expressed stem cell markers and to compare the protein expression profile of normal goat cornea with damaged and reconstructed corneal epithelium, the resultant cells and corneas were collected, fixed in 4% paraformaldehyde, and embedded in tissue Tek OCT compound (Electron Microscopy Sciences, Washington, PA) for sectioning. For hematoxylin and eosin (H&E), PAS, and AB-PAS staining, slides were re-fixed, washed, and stained by their respective methods. For immunofluorescent staining, slides were re-fixed and rinsed three times (15 min each time) in PBS that contained 0.5% Triton-X 100 and then incubated with 2% BSA in PBS for 60 min at 37 °C. Primary antibodies (for sources and working dilutions, see Table 1) were applied to tissues for 2 h at 37 °C. After washing, fluorescein-conjugated secondary antibodies were applied for 30 min at 37 °C. Immunofluorescence microscopy was performed with a Nikon microscope (Nikon, Tokyo, Japan).

**Figure 1:**
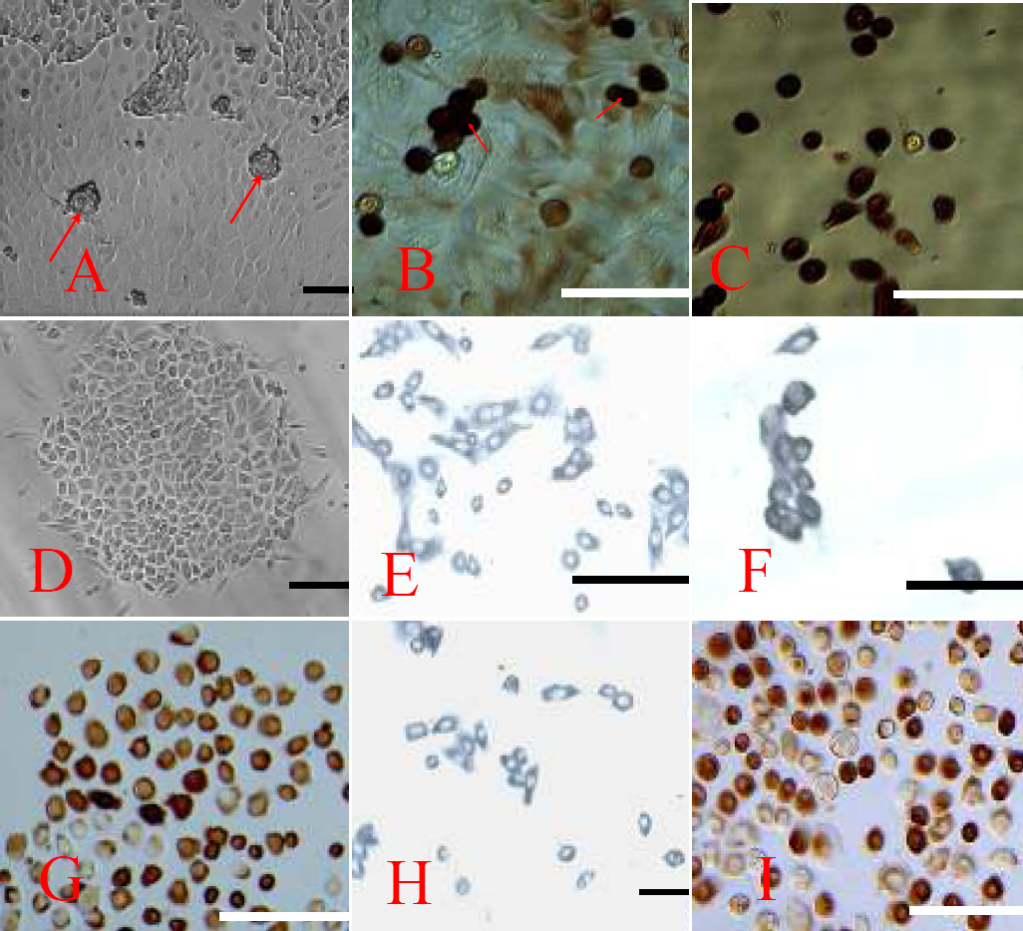
The characteristics of epidermal adult stem cells. **A**: Primary EpiASC clones derived from goat ear skin formed cell clusters, 100X. **B**: Clusters of primary EpiASC positively express Oct4 (9E3), 200X. **C**: Single primary EpiASC express Oct4 (9E3), 200X. **D**: Subcultured EpiASC formed holoclones in vitro, 100X. **E**: Subcultured EpiASC express CK19 (A53-B/A2), 200X. **F**: Subcultured EpiASC express P63 (4A4), 200X. **G**: Subcultured EpiASC express β_1_-integrin (W1B10), 200X. **H**: Subcultured EpiASC express CD34, 100X. **I**: Part of the subcultured EpiASC expresses Oct4, 200X. The scale bar represents 100 μm.

Immunocytochemistry staining was done by the PV-6002 Histostain™–Plus method, using kits from Beijing Zhongshan Biological and Technological Co. (Beijing, China). Sections were blocked with normal goat serum for 15 min at room temperature and incubated with primary antibodies (Table 1) overnight at 4 °C. Cells were rinsed with 0.1 M PBS without calcium and magesium and incubated with the secondary goat anti-mouse IgG for 15 min at 37 °C. The samples were washed and then incubated with the streptavidin/peroxidase work medium (S-A/HRP) for 15 min at 37 °C. Cells were rinsed in 0.1 M PBS without calcium and magesium, and the samples were finally incubated with the 3, 3′-diaminobenzidine (DAB) peroxidase substrate to give a brown stain and counterstained with hematoxylin or with chromogen solution, BCIP/NBT, to give a blue stain. After washing with PBS, the mounted sections were examined and photographed with an epifluorescent microscope, (Nikon, Tokyo, Japan).

## Results

### The characteristics and origin of epidermal adult stem cells

**Figure 2:**
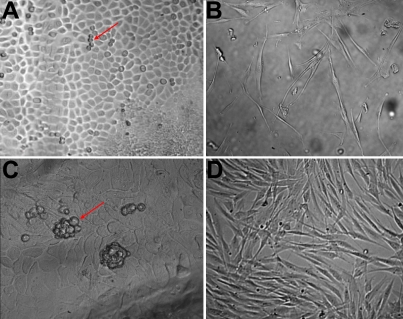
Morphological properties of cells derived from the epidermis are distinct from cells derived from the dermis. **A**: After about 3 to 4 days in culture, cells derived from the epidermis expand in a mosaic way, self renewing cells appeared on the surface of the cell layer (red arrow). **B**: Shows fibroblast cells derived from the dermis. **C**: After about 8 to 10 days, stem-like cell clones formed on the surface of the epithelial cell layer (red arrow). **D**: After 8~10 days fibroblasts derived from the dermis became confluent and stem-like cell clones were not observed.

We first cultured skin explants in a serum-based medium. After five to six days in culture, we found epithelial-like cells growing in a mosaic pattern surrounding the explant. Next, small, blast-like cells with a high nuclear/cytoplasmic ratio appeared on the surface of the epithelial-like cells. After 12–15 days, these cells formed clones with diameters of approximately 50–100 μm (Figure 1A). Immunohistochemistry showed that these primary EpiASCs either in clusters or single were Oct4 positive (Figure 1B,C). These clones were picked under the dissecting microscope, trypsinized into single-cell suspensions, and cultured in a serum-free medium on gelatin-coated dishes. Subsequently, the adherent cells formed holoclones (Figure 1D). These clones were expanded in vitro. Immunohistochemistry showed that the holoclone cells strongly expressed CK19, P63, β1-integrin, and CD34, a group of previously reported EpiASC markers (Figure 1E-H). Surprisingly, we found ~50%of subcultured EpiASC still strongly expressing Oct4 (Figure 1). To confirm the origin of isolated EpiASC, we separated the epidermis from the dermis by dispase II treatment and cultured both of them in the same medium. After three to four days, we found that cells that were derived from the epidermis expand in a mosaic pattern (Figure 2A), which is different from the fibroblast cells derived from dermis (Figure 2B). After 8–10 days, cell clones were formed in the surface of epithelial cells, which exhibited the same properties as stem-like cell clones from total skin explant culture (Figure 2C). In contrast, the fibroblast cells became confluent, and no stem-like cell clones were observed (Figure 2D). These results showed that EpiASC are derived from the epidermis of the skin.

### Human amniotic membrane serves as a niche for epidermal adult stem cells

**Figure 3:**
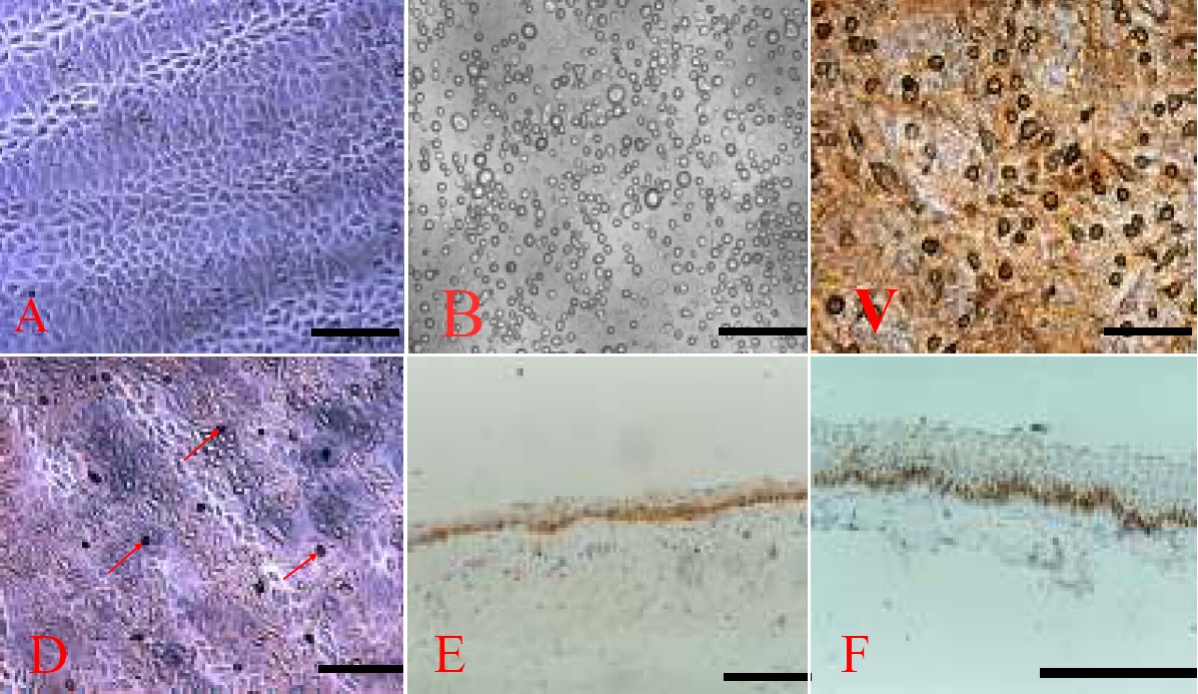
Human amniotic membrane serving as a niche for epidermal adult stem cells and the formation of EpiASC-HAM sheet. **A**: Passage 3 epidermal stem cell was used as seed cell to reconstruct artificial corneal epithelium, 100X. **B**: EpiASC cultured on HAM for 30 min, 100X. **C**: EpiASC cultured on HAM for 20 days and differentiated into keratinocytes expressing CK1/10 (ab20121), 100X. **D**: P63-positive EpiASC surrounded by P63-negative cells, showing EpiASC reconstructing a niche on HAM, 100X. **E**: P63-positive pattern of ESH, 200X. **F**: P63 (4A4) expression in a wavy pattern along the basal membrane of normal corneal limbus, 200X. The scale bar represents 100 μm.

Preserved HAM transplantation alone was previously shown to be sufficient to reconstruct the corneal surface in cases of partial LSCD [31,32], suggesting that HAM may help expand the residual limbal epithelial stem cells. Clinical and experimental studies have also shown that ex vivo-expanded limbal epithelial cells on HAM are capable of restoring the corneal surface in LSCD [32]. Meller et al. [33] provided evidence that HAM cultures preferentially preserve and expand limbal epithelial stem cells that maintain their in vivo properties of slow cycling, label retention, and undifferentiation. We proposed that HAM might be an ideal matrix for ex vivo preservation and expansion of EpiASC. To investigate this, we seeded EpiASC that were isolated from a single cell (Figure 3A) on denuded HAM (Figure 3B). The culture was submerged in a serum-free medium for one week and then exposed to air by lowering the medium level over two weeks. We found that EpiASC adhered to HAM within 20–30 min, forming EHS. EpiASC remained quiescent during the first two days and then proliferated quickly during a further three days culture. We also observed that EpiASC formed clones in a similar way when co-cultured with 3T3 cells [data not shown]. To promote EpiASC proliferation, we exposed the surface of EHS to the air a week after culture and added 50 μg/ml Vitamin C to the medium. We found that EpiASC proliferation was arrested and differentiation had begun. The surface of EHS was covered with flattened cells, which expressed CK1/10 (Figure 3C). When the EHS was digested with 0.25% trypsin for 20–25 min, we found that a single cell layer was firmly attached to the HAM. Staining with anti-P63 antibody revealed that P63-positive cells were surrounded by groups of P63-negative cells (Figure 3D). This suggested that in the serum-free culture conditions, EpiASC reconstructed their niche on the surface of HAM. Immunostaining also showed that the P63 expression pattern of the EHS (Figure 3E) was similar to the pattern observed within the basal membrane layer of corneal epithelium (Figure 3F), further evidence that HAM can serve as a niche for EpiASC and can support EpiASC proliferation in vitro.

### Clinical results of transplantation of EpiASC-HAM sheet to the corneal surface

**Figure 4:**
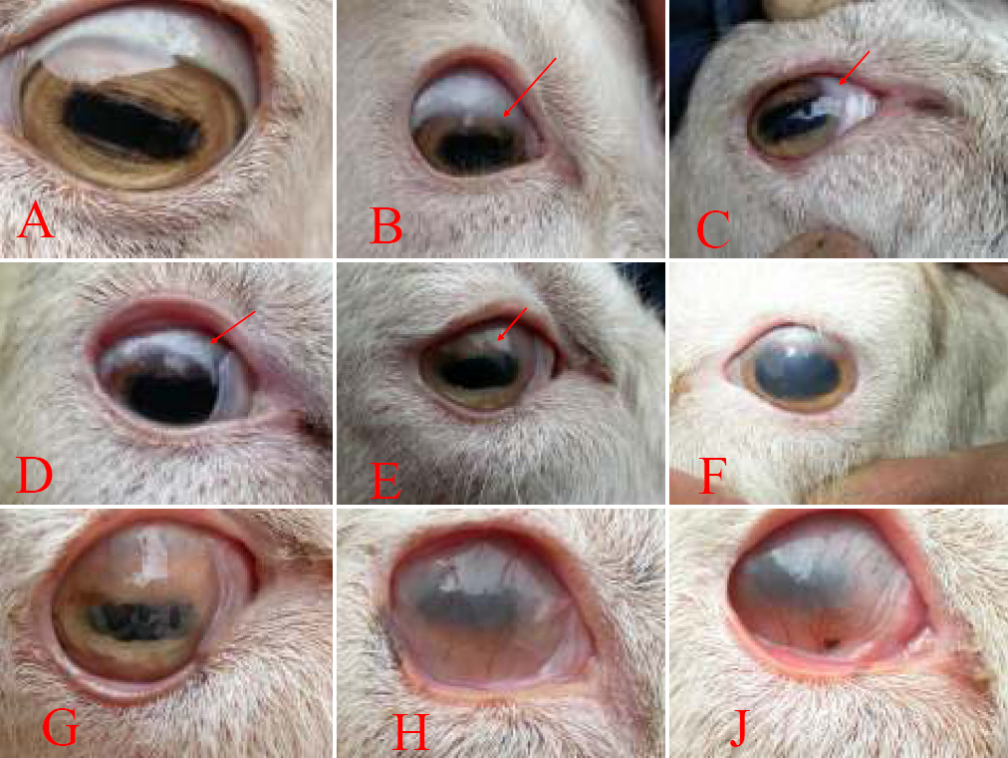
Clinical results of transplantation of EpiASC-HAM sheet to the damaged corneal surface. **A**: Untreated goat eye is showing a normal cornea. **B–D**: Three of the experimental eyes regained four quadrants of clear cornea and were deemed successful reconstructions. **E-G**: Three of the experimental eyes regained three quadrants of clear cornea and were deemed partial successes for reconstruction. **H**: The reconstructed ocular surface of total LSCD without transplantation was covered with vascularized pannus. **I**: The reconstructed ocular surface of total LSCD with HAM only transplantation was also covered with vascularized pannus.

We have previously reported the phenotypic outcome of transplantation of EHS to the corneal surface of total LSCD with a follow-up period of 24 months [28]. During this study, with an average follow-up period of 30 months, approximately 30% (3/10) of the eyes of treated animals regained four quadrants of clear cornea. This was assessed as a success according to the criterion of Ti et al. [29]. However, part of the limbus of these three eyes was unclear, which is distinct from normal eyes (Figure 4A-D). Half (5/10) of the eyes of the treated animals regained three quadrants of clear cornea, which was deemed a partial success [29], but the further limbus of these eyes was opaque (Figure 4E-G). The remaining 20% (2/10) failed to regain two quadrants of clear cornea (data not shown). The corneas of the control eyes were covered with pannus (Figure 4H,I), which manifested as vascularization and chronic inflammation of the cornea, ingrowth of fibrous tissue, and corneal opacification.

### Epidermal adult stem cells can be converted into corneal epithelium by corneal stroma

**Figure 5:**
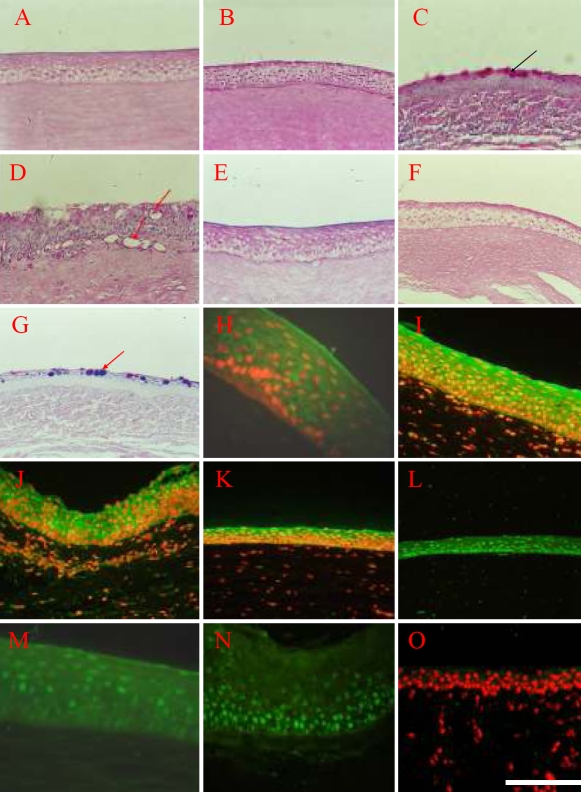
Epidermal adult stem cells can be converted into corneal epithelium by corneal stroma. **A**: PAS staining of the normal cornea epithelium showed no goblet cells, 200X. **B**: PAS staining of the reconstructed cornea epithelium also show no goblet cells, 200X. **C-D**: PAS staining shows the invaded goblet cells in the corneal surface of control group, 200X. The black arrow in **C** is pointing to the invaded goblet cells, the red arrow in **D** is pointing to the blood vessels. **E**: Normal cornea is shown, and the AB-PAS staining is red, characteristic of neutral mucus, 200X. **F**: The reconstructed cornea is shown, and the AB-PAS staining is also red, characteristic of neutral mucus, 200X. **G**: AB-PAS staining show goblet cells as purple, which is characteristic of mixed mucus, 200X. The red arrow in **G** is pointing to the goblet cells in the corneal epithelium of control group which were stained purple by the AB-PAS staining method. The results of immunohistochemistry showed that normal cornea epithelium (**H**) expressed CK3 (I.BB.787), 400X, as well as reconstructed cornea epithelium (**I**) expressed CK3, 400X. Both normal cornea epithelium (**J**) and reconstructed cornea epithelium (**K**) expressed CK12 (SC-17099) which is a specific marker for differentiated corneal epithelium, 400X. Furthermore, both of normal cornea epithelium (**L**) and reconstructed cornea epithelium (**M**) expressed PAX-6 (AB5409), 400X. Although the reconstructed epithelium of control group (**N**) expressed PAX-6 (AB5409), it did not express CK3 (**O**), showing that this kind of epithelium derived from the control group did not share the character with normal corneal epithelium, 200X. The scale bar represents 100 μm.

We have previously reported that autologous EpiASC transplantation reconstructs the ocular surface of total LSCD to functionality [28], which only involves the morphological and histological features of the reconstructed ocular surface. However, the fate of grafted EpiASC after transplantation was not studied. In the present study, we compared the features of normal corneal epithelium (NCE), reconstructed cornea epithelium (RCE), and the pannus (P) of control groups by H&E, PAS, and immunocytochemistry staining. The results showed that reconstructed cornea shares certain features with normal cornea such as the corneal surface was smooth and the epithelium was integral without goblet cells (Figure 5A,B). In contrast, the corneal surface in the control group was covered with fibers, blood vessels, and goblet cells (Figure 5C,D). Interestingly, AB-PAS staining showed that both normal and reconstructed corneal epithelium were red (Figure 5E,F), which is characteristic of neutral mucus. In contrast, the goblet cells in the control group were purple (Figure 5G), characteristic of mixed mucus. These findings suggest that the corneal epithelium of reconstructed cornea has a function of secretion similar to normal corneal epithelium and distinct from invading goblet cells.

To confirm whether EpiASC may serve as LSCs to functionally reconstruct the damaged cornea of LSCD, a series of markers of corneal epithelium were compared among NCE, RCE, and P. The results showed that the RCE and NCE exhibit similar expression profiles of CK3, CK12, and PAX-6 (Figure 5H-M). The pannus expressed PAX-6 (Figure 5N) but not CK3 (Figure 5O). These data provide good evidence that EpiASC regenerated the function of damaged corneal surface caused by total LSCD.

**Figure 6:**
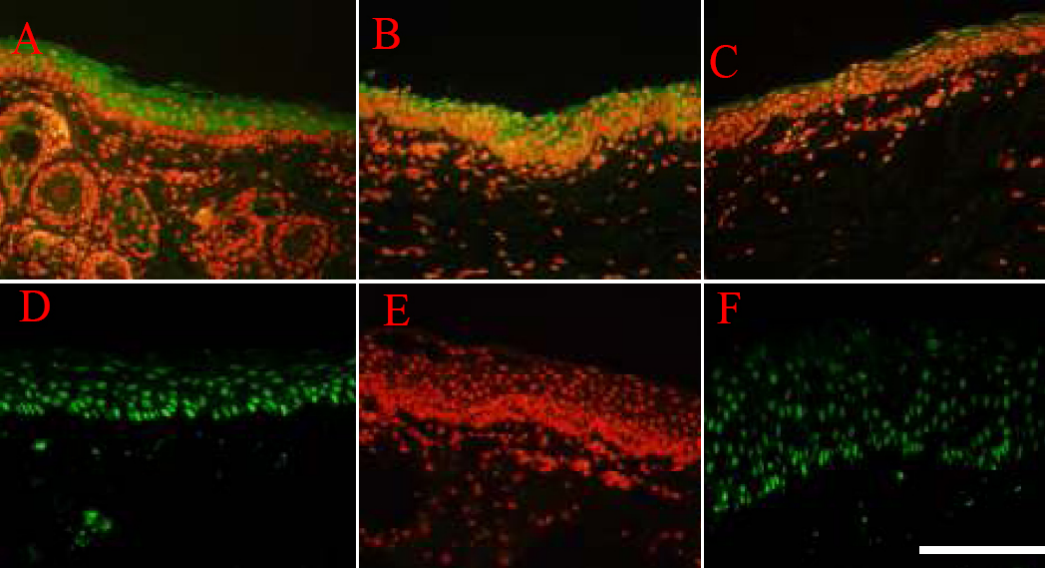
The expression profile of CK1/10 and PAX-6 in normal skin and reconstructed cornea epithelium six months after the operation and 12 months after the operation. **A**: CK1/10 (ab20121) was highly expressed in the cell cytoplasm of superficial epithelial cells of NS, 200X. **B**: CK1/10 (ab20121) was weakly expressed in the cell of reconstructed limbal epithelial basal layer six months after transplantation, 200X. **C**: CK1/10 (ab20121) was weakly expressed in the cell cytoplasm of RCE superficial epithelial cells six months after transplantation, 200X. **D**: PAX-6 (AB5409) was expressed in the nucleus of epithelial cells of RCE six months after the operation, 200X. **E**: CK1/10 (ab20121) was not expressed by RCE 12 months after the transplantation, 200X. **F**: PAX-6 (AB5409) was expressed in the nucleus of epithelial cells of RCE 12 months after the operation, 200X. The scale bar represents 100 μm.

Although we could not provide direct evidence that the reconstructed corneal epithelium were derived from grafted EpiASC (because of the lack of a valid marker to trace the fate of EpiASC during host wound repair), there is indirect evidence suggesting that the reconstructed corneal epithelium derives from grafted EpiASC. To show this, the expression profiles both of CK1/10 and PAX-6 were compared in normal skin (NS) and RCE six months after the operation and 12 months after the operation. We found that the expression of CK1/10 in NS is much greater than in RCE six months after operation (Figure 6A-B); however, PAX-6 was strongly expressed in the nucleus of epithelial cells in the RCE (Figure 6C). Interestingly, CK1/10 was also expressed in the cell cytoplasm of limbal and central corneal epithelial basal layer, which is different from the normal location of CK1/10 in NS (Figure 6D). NS is typically expressed in the superficial epithelial cells. Furthermore, CK1/10 was not expressed by the RCE 12 months after the operation (Figure 6E) while PAX-6 was strongly expressed in the nucleus of epithelial cells of the RCE (Figure 6F). This suggests that the expression of CK1/10 was down-regulated by the corneal stroma of the host animal during the process of corneal epithelium regeneration and that PAX-6 expression was upregulated.

## Discussion

For treatment of severe ocular surface diseases such as SJS and OCP, it is crucial to find a strategy that avoids the risks of allogeneic immunorejection and immunosuppression. Recent progress in this field is encouraging and suggests that autologous oral mucosal epithelial cells [34-36], mesenchymal stem cells [37], and conjunctival epithelial cells [38] may serve as alternative seed cells for treating these severe ocular surface diseases. The present study shows that tissue-engineered cell sheets composed of adult epidermal stem cells can successfully reconstruct the damaged cornea of total LSCD in goats. Ten LSCD goats (10 eyes) were treated with this approach, leading to the restoration of corneal transparency and improvement of postoperative visual acuity to varying degrees in 80% (8/10) of the experimental eyes. In comparison, the corneal epithelium of two control groups either with HAM transplantation only (n=8) or without any transplantation (n=8) showed irregular surfaces, diffuse vascularization, and pannus on the entire cornea. The reconstructed corneal epithelium expressed CK3, CK12, and PAX-6 and had the function of secreting glycocalyx-like material (AB-PAS positive). During the follow-up period, all corneal surfaces remained transparent and there were no serious complications.

The ocular surface of either human or animal origin exposed outside of the body is suitable to observe and evaluate the outcome of transplant experiments. This fact along with the well defined locations of different cell types within the cornea provides a useful model for studying the plasticity of adult stem cells. EpiASC as well as other adult stem cells readily lose their stemness during their propagation in vitro. To overcome this problem, we developed a serum-free medium containing fibroblast cell-conditioned medium to culture EpiASC, and we selected HAM as a substrate to carry EpiASC for fabricating ESH. Many studies have shown that HAM can serve as a niche for limbal stem cells to maintain their stemness in vitro [32,39]. Our own results showed that P63-positive cells were strictly located to the basal layer of the ESH on day 21 of culture and suggested that HAM is a suitable carrier for EpiASC to maintain stemness.

During the process of repairing the damaged ocular surface of LSCD in goat, the CK1/10 marker was down-regulated by corneal stroma. This is indirect evidence that EpiASC can survive in the host tissue and can be induced into corneal epithelium in vivo, which expresses CK3, CK12, and PAX-6. These results also suggest that the probable mechanism of EpiASC reconstruction of the damaged ocular surface of total LSCD involves the down-regulation of CK1/10 and upregulation of PAX-6 by the factors derived from corneal stroma. *PAX-6* is a master gene that controls eye formation during embryonic development [40,41]. Pearton et al. [14] suggested that transdifferentiation of epithelium into epidermis occurs through a multi-step process that is triggered by dermal developmental signals, which involves the down-regulation of *PAX-6*, loss of expression of corneal-specific keratins, and induction of basal keratinocyte markers [14]. This evidence supports the proposal that *PAX-6* plays a crucial role in eye development [40,41]. More work is needed to monitor how and when the silenced *PAX-6* in EpiASC is reactivated and the *CK1/10* gene is down-regulated by the corneal stroma. In turn, this work will shed light on the reconstruction mechanism of the damaged ocular surface of total LSCD by EpiASC transplantation.
